# Sulforaphane as a potential modifier of calorie-induced inflammation: a double-blind, placebo-controlled, crossover trial

**DOI:** 10.3389/fnut.2023.1245355

**Published:** 2023-11-28

**Authors:** Hidde P. van Steenwijk, Anna Vinken, Frits H. M. van Osch, Herman Peppelenbos, Freddy J. Troost, Aalt Bast, Khrystyna O. Semen, Alie de Boer

**Affiliations:** ^1^Food Claims Centre Venlo, Campus Venlo, Faculty of Science and Engineering, Maastricht University, Venlo, Netherlands; ^2^Department of Clinical Epidemiology, VieCuri Medical Center Venlo, Venlo, Netherlands; ^3^Department of Epidemiology, NUTRIM, Faculty of Health, Medicine and Life Sciences, Maastricht University, Maastricht, Netherlands; ^4^Department of Food Innovation, HAS University of Applied Sciences, 's-Hertogenbosch, Netherlands; ^5^Division of Gastroenterology-Hepatology, Department of Internal Medicine, School of Nutrition and Translational Research in Metabolism, Maastricht University Medical Center+, Maastricht, Netherlands; ^6^Food Innovation and Health, Center for Healthy Eating and Food Innovation, Maastricht University, Maastricht, Netherlands; ^7^Faculty of Science and Engineering, University College Venlo, Campus Venlo, Maastricht University, Venlo, Netherlands; ^8^Department of Pharmacology and Toxicology, Faculty of Health, Medicine and Life Sciences, Maastricht University, Maastricht, Netherlands

**Keywords:** sulforaphane, glucoraphanin, phenotypic flexibility, hormesis, biomarkers, nutrients, diet, inflammation

## Abstract

**Background and aims:**

Observational data indicate that diets rich in fruits and vegetables have a positive effect on inflammatory status, improve metabolic resilience and may protect against the development of non-communicable diseases. Nevertheless, experimental evidence demonstrating a causal relationship between nutrient intake (especially whole foods) and changes in metabolic health is scarce. This study investigated the pleiotropic effects of sulforaphane from broccoli sprouts, compared to pea sprouts, on biomarkers of endothelial function, inflammation and metabolic stress in healthy participants subjected to a standardized caloric challenge.

**Methods:**

In this double-blind, crossover, randomized, placebo-controlled trial 12 healthy participants were administered 16 g broccoli sprouts, or pea sprouts (placebo) followed by the standardized high-caloric drink PhenFlex given to disturb healthy homeostasis. Levels of inflammatory biomarkers and metabolic parameters were measured in plasma before and 2 h after the caloric overload.

**Results:**

Administration of broccoli sprouts promoted an increase in levels of CCL-2 induced by caloric load (*p* = 0.017). Other biomarkers (sICAM-1, sVCAM-1, hs-CRP, and IL-10) individually showed insignificant tendencies toward increase with administration of sulforaphane. Combining all studied biomarkers into the systemic low-grade inflammation score further confirmed upregulation of the inflammatory activity (*p* = 0.087) after sulforaphane. No significant effects on biomarkers of metabolic stress were detected.

**Conclusion:**

This study has demonstrated that sulforaphane facilitated development of a mild pro-inflammatory state during the caloric challenge, which could be suggestive of the onset of the hormetic response induced by this phytonutrient. The use of integrative outcomes measures such as the systemic low-grade inflammation score can be viewed as a more robust approach to study the subtle and pleiotropic effects of phytonutrients.

**Clinical trial registration:**www.clinicaltrials.gov, identifier NCT05146804.

## 1 Introduction

Non-communicable diseases (NCDs) are the leading cause of death worldwide, accounting for 71% of total deaths each year (1). Chronic low-grade inflammation (CLGI) plays a crucial role in the pathology of NCDs, but also appears to affect apparently healthy people as a consequence of poor lifestyle choices, e.g., overeating, smoking and excessive alcohol consumption (2–4). A wealth of observational data indicates that healthy lifestyle choices, such as moderate exercise and diets rich in fruits and vegetables, have a particularly positive effect on inflammatory status and the development of various NCDs (5–7). Nevertheless, randomized placebo-controlled trials frequently fail to demonstrate causal relationships between nutrient intake (especially whole foods) and changes in metabolic health (8–11).

With an increasing understanding of disease, health is no longer seen as simply a fixed entity of complete physical, mental, and social well-being, but redefined as our body’s ability to cope with everyday challenges (11–15). The concept of this phenotypic flexibility implies that health can be measured by the ability to adapt to conditions of temporary stress. Challenge testing, which may involve exercise or caloric overload, is often used in practice to assess phenotypic flexibility. This may be a more sensitive way of assessing the effects of fruits and vegetables on the health status of the healthy low-risk population (14–21). Unlike drugs, food-derived compounds exert subtle effects in the general population rather than treating specific disease states in patients (12, 13). While pharmacology is still dominated by the “one disease—one target—one drug” paradigm, nutritional interventions frequently work on many pathways involved in the development of chronic diseases, with hormetic principles at its heart (11–14, 22, 23). Moreover, nutritional science (and within claims substantiation) often still focuses on this more pharmacological approach, so that it is only considered ‘effective’ if one nutrient affects one target (24). All things considered, it is challenging to measure beneficial effects in healthy people, and especially when you are trying to see the effect of one nutrient on one target. It is assumed that any intervention works via a hormetic mechanism if the final beneficial effect on phenotypic flexibility is in fact achieved through initial structural damage or functional overstrain, which is ultimately responsible for the activation of the protective mechanisms (25–37). For example, physical activity and mild stress-inducing phytonutrients called hormetins are known to increase levels of oxidative stress, but this appears to be beneficial for health (11, 22, 38–41). Most dietary hormetins are known to induce the expression of antioxidant enzymes by triggering a pro-oxidant response via activation of the nuclear factor E2-related factor 2 (Nrf2)-pathway (42–50). While the degree of immediate hormetic effects following exposure to a particular stress may be only moderate, the chain of events following the initial phase leads to biologically amplified effects that are much larger, synergistic, and pleiotropic and therefore require integrative approach to assessment of the outcomes (12, 13, 16, 34, 38, 40). Norde et al. (51) proposed a novel approach to measure CLGI by combining multiple biomarkers into a systemic low-grade inflammation score.

Glucoraphanin, the biogenic precursor of sulforaphane, is present in large amounts in broccoli sprouts (52, 53). After damage to plant tissue, e.g., through chewing, glucoraphanin comes into contact with the enzyme myrosinase, which is separated from its substrate in the intact vegetable, and subsequently is converted to sulforaphane (53–55). Sulforaphane is the most potent naturally occurring inducer of Nrf2 (42–48). Previous studies showed that long-term consumption of broccoli sprouts improved fasting blood glucose levels and stabilization of insulin response in type 2 diabetic patients, particularly obese patients (56, 57). To our knowledge, no experimental study has been conducted to investigate the effects of broccoli sprouts on integrative outcome measures. In the current study, we investigate the pleiotropic effects of broccoli sprouts, compared to pea sprouts, on biomarkers of endothelial function, inflammation and metabolic stress in healthy participants subjected to a standardized caloric challenge.

## 2 Methods

We have conducted a randomized, placebo-controlled, double-blind study with a cross-over design (58, 59). The study protocol (NL77272.068.21) was approved by the Medical Ethics Review Committee of Maastricht University Medical Centre+ (MUMC+) and Maastricht University, Maastricht, the Netherlands, and performed in full accordance with the declaration of Helsinki of 1975 as revised in 2013, Fortaleza, Brazil (60). The trial registration number within ClinicalTrials.gov is NCT05146804. All subjects provided written informed consent.

### 2.1 Subjects

Twelve healthy participants (11 males and one female) were recruited by local and social media advertisements. Inclusion criteria were that participants were between 18 and 50 years old, had a body mass index (BMI) between 18.5 and 30 kg/m^2^, with a stable weight (<5% body weight change) and constant eating habits over the past 3 months. Exclusion criteria were the previous diagnosis of an inflammatory condition or disease or a history of hypothyroidism, chronic kidney or/and liver disorders, coronary artery disease, malignant hypertension, seizures, involved in intensive sports activities more than four times a week or at top sport level, regular intake of medication that may affect inflammatory response including NSAIDs, psychotic, addictive, or other mental disorders, aversion, intolerance or allergy to cruciferous vegetables and/or palm olein, dextrose, protein supplement, vanilla aroma, the use of dietary supplements with potential effects on antioxidant or inflammatory status and/or viral or bacterial infections requiring the use of antibiotics, laxatives and anti-diarrheal drugs 4 weeks prior to inclusion, excessive alcohol consumption (≥28 consumptions approx. 250 g alcohol per week), pregnancy and/or breastfeeding, reported slimming or medically prescribed diet, as well as adhering to a vegetarian or vegan lifestyle. The sample size calculation is based on a crossover study by Meijer et al. (61) [Trial NL3290 (NTR3435)] in which they examined whether broccoli seedlings could reduce glucose-induced postprandial inflammation in healthy male participants. Meijer et al. measured plasma concentrations of sVCAM-1 and sICAM-1 (primary outcomes) at different timepoints in healthy men after consumption of broccoli seedlings or lettuce (placebo). The detectable difference used for the sample size calculation is calculated based on the mean concentrations of sVCAM-1 (ng/mL). Variance = Mean difference/SD Variance = (26.7–2.4)/14.20 = 1.711.

‘Variance explained by special effect’ was set to 0.5. We calculated an effect size of 0.54. A power of 80% was implemented, the chance of having a type I error was 5% and an effect size of 0.54 (medium effect size). In present study, we have two groups (two repeated measures, within-between interaction) yielding 10 participants, and considering a 20% dropout, which results in the final sample size of 12 participants.

### 2.2 Study design and procedures

**Figure 1:**
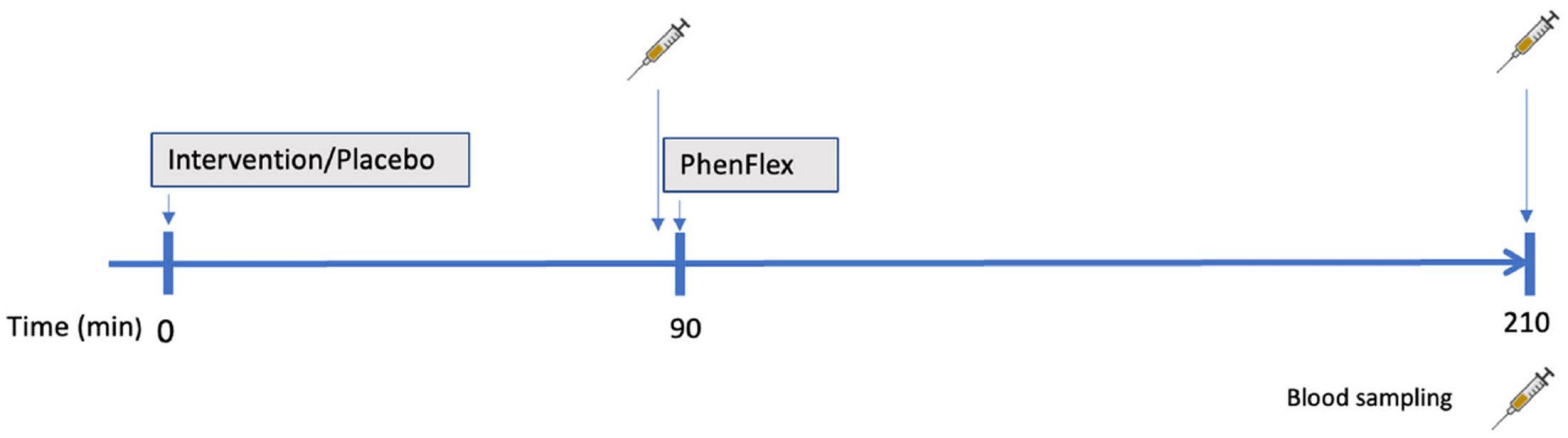
Schematic presentation of a study visit. Administration of intervention (sulforaphane/placebo) was followed in 90 min by administration of standardized caloric challenge PhenFlex. Blood samples were obtained before (90 min) and 2 h after (210 min) PhenFlex challenge.

Commercially available broccoli sprouts BroccoCress®, a rich source of glucoraphanin (the parental glucosinolate of sulforaphane), were used as the experimental product. In total, 16 g of sprouts were utilized, equivalent to one serving (portion) of microgreens. Sulforaphane (BroccoCress®) and the placebo (Affilla Cress®) were randomly administered to each participant on separate testing days (as detailed in section 2.3). The period between two visits was 7 ± 3 days. Information about demographics, alcohol consumption, and anthropometric data were assessed on the first visit. Body mass index (BMI), total body fat and visceral fat were measured using the Omron BF511R® monitor. The same testing scheme was applied during two visits (Figure 1), i.e., each participant received a single serving of intervention/placebo, which after 90 min was followed by oral administration of the PhenFlex challenge. Blood samples were collected twice, just before intake of PhenFlex and 120 min after. All participants were instructed to come fasted to each visit, to avoid consumption of broccoli or other cruciferous vegetables 2 days before the visit and to restrain from intense physical activity on the day of the visit. During the visit, participants remained in the testing location and were allowed to drink water *ad libitum*. No food intake was permitted during the visit.

### 2.3 Intervention and caloric challenge (PhenFlex)

The broccoli sprouts were cut approximately 1 cm below the leaves (with the hypocotyl being cut below the cotyledons), weighed, and mashed with a small amount of tap water (approximately 13°C) in a kitchen blender for 30s at room temperature immediately before administration (Premium Impuls Blender Smoothiemaker; Impuls; 180 W). Subsequently, tap water (approximately 13°C) was added to the mixture to bring the total amount to 250 mL and participants were instructed to drink the entire mixture immediately. Commercially available pea sprouts (Affilla Cress®) were used as a placebo in this study since pea sprouts do not contain glucoraphanin/sulforaphane. Affilla Cress (16 g) was prepared and administered in a similar fashion. Blinding of participants was ensured by the even appearance of both drinks and the use of nasal plugs during the consumption of the investigational products. The placebo or intervention product was prepared by a researcher who was not involved in any other study procedures and data analysis. Ninety minutes after administration of the investigational products, participants were asked to drink the high-fat, high-glucose, high-caloric product (PhenFlex) (62). For the preparation of the PhenFlex (400 mL, 950 kcal) 60 g palm olein, 75 g dextrose, 20 g protein, 0.5 g artificial vanilla aroma and 320 mL tap water were used (62). In all cases, PhenFlex mixtures were freshly prepared, and the participants were instructed to consume the drink within 5 min.

### 2.4 Blood sampling and assessment of biomarkers

Samples of venous blood were taken twice per visit from the antecubital vein for measurement of inflammatory and metabolic biomarkers. Samples were collected in 4 mL BD tubes containing K2EDTA as anticoagulant, and centrifuged for 5 min (at 3,000 g, 4°C) within 30 min after collection. Plasma was stored at ≤−80°C until the day of analysis.

Plasma samples were analyzed for inflammatory biomarkers, sVCAM-1, sICAM-1, IL-1β, IL-6, TNF-α, CCL-2, IL-8, IL-10, adiponectin, hs-CRP, and IL-12 p70 using Enzyme-linked immunosorbent assays (R&D Systems Netherlands; Supplementary Table 1). A systemic low-grade inflammation score was generated summing the z-score log-transformed inflammatory biomarkers plasma concentration (sICAM-1, sVCAM-1, IL-6, TNF-α, CRP, IL-12, CCL-2, IL-1β, and IL-8). The z-score log-transformed plasma adiponectin and IL-10 levels were subtracted from the systemic low-grade inflammation score due to their anti-inflammatory properties (51). Plasma samples were analyzed for glucose via colorimetric assay (Cayman Chemical; Glucose Colorimetric Assay Kit, Item No. 10009582) and lipoprotein A using Enzyme-linked immunosorbent assay (Abcam®; Human Lipoprotein A SimpleStep ELISA® Kit, ab212165).

### 2.5 Statistical analysis

All normally distributed data are presented as mean ± standard deviation (SD). The non-normally distributed data are shown as median (interquartile range). For categorical variables, frequency and/or percentages are presented. Differences between the groups were assessed by paired sample T-tests for normally distributed parameters, or Wilcoxon signed-rank tests for the data that was not normally distributed. Wilcoxon signed-rank tests were performed to check for potential carryover effects. Associations between clinical features and biomarkers were assessed using Pearson (normally distributed parameters) or Spearman’s rank (non-normally distributed) correlation coefficient. All analyses were performed, two-tailed with a *p* ≤ 0.05 considered statistically significant.

## 3 Results

### 3.1 Subject characteristics

**Figure 2:**
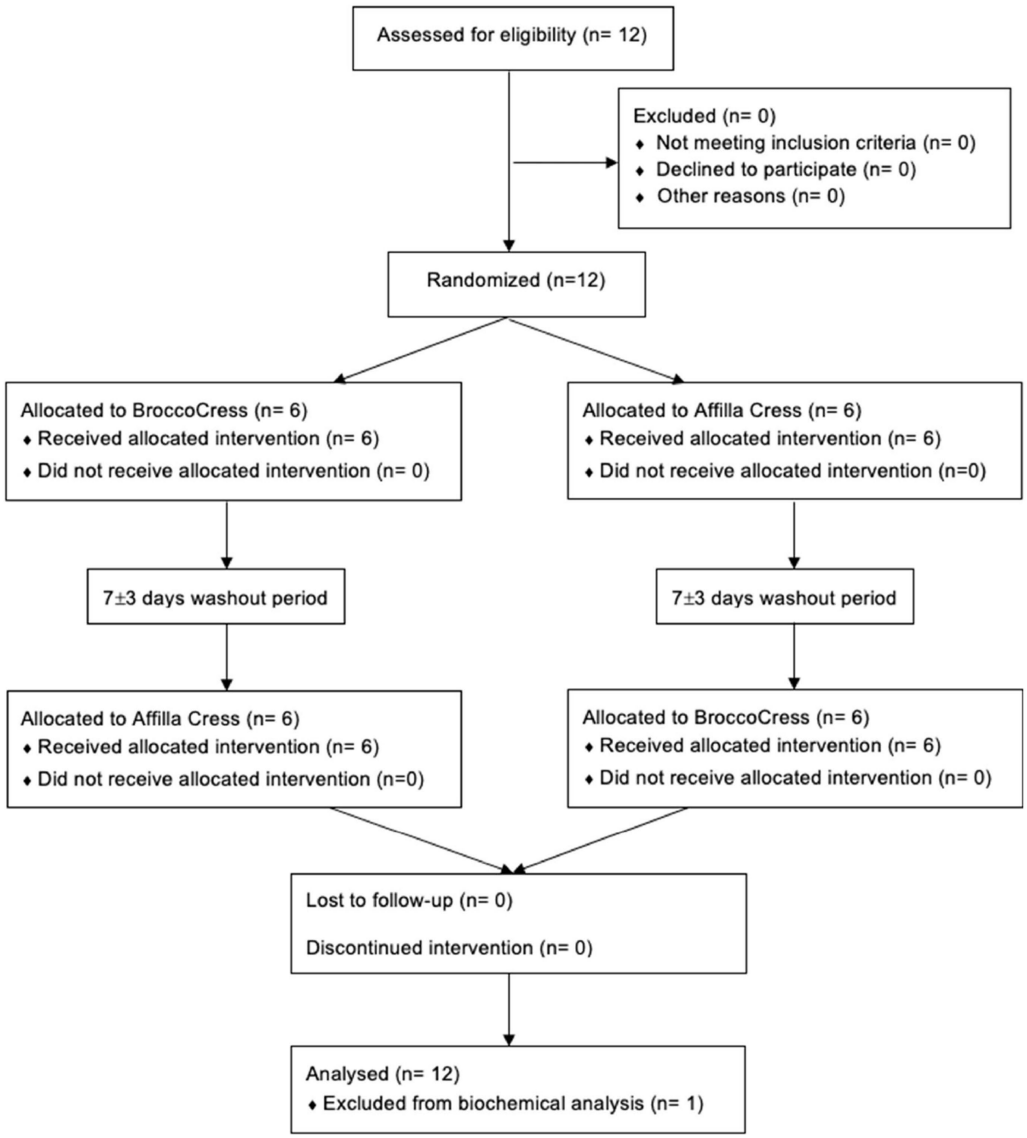
CONSORT flow diagram.

Between November 2021 and January 2022, a total of 12 subjects were enrolled into the study and randomly allocated to either initial administration of sulforaphane or placebo. Baseline characteristics of the study population (*n* = 12) are summarized in Table 1. All participants completed the study and were included in the data analysis for biochemical testing (Figure 2). The pre-test (Wilcoxon rank sum test) revealed no differences between treatment allocations for all parameters (all *z* < 0.00, *p* > 0.14). Absence of sulforaphane in placebo was verified by testing urine samples which showed significantly higher concentration of the total metabolites of sulforaphane after intake of broccoli sprouts (8.2 vs. 0.4 μmol, *p* < 0.001).

**Table 1:** Characteristics of the study participants.

Characteristics	Population (*n* = 12)
**Sex (*n*, %)**	
Female	1 (8.3)
Male	11 (91.7)
Age [years, mean (SD)]	26.9 (3.6)
BMI (kg/m^2^)	23.1 (1.6)
**Body fat (%)**	
Female	28.9 (n/a)
Male	21.4 (3.1)
All	22.0 (3.6)
Visceral fat level [mean (SD)]	5.17 (1.57)
**Alcohol consumption, *n* (%)**	
Moderate	0 (0)
Heavy	9 (75)
Very heavy	3 (25)
**Smoking status, *n* (%)**	
Smoker	5 (42)
Non-smoker	7 (58)

### 3.2 The effect of sulforaphane on endothelial activation

**Figure 3:**
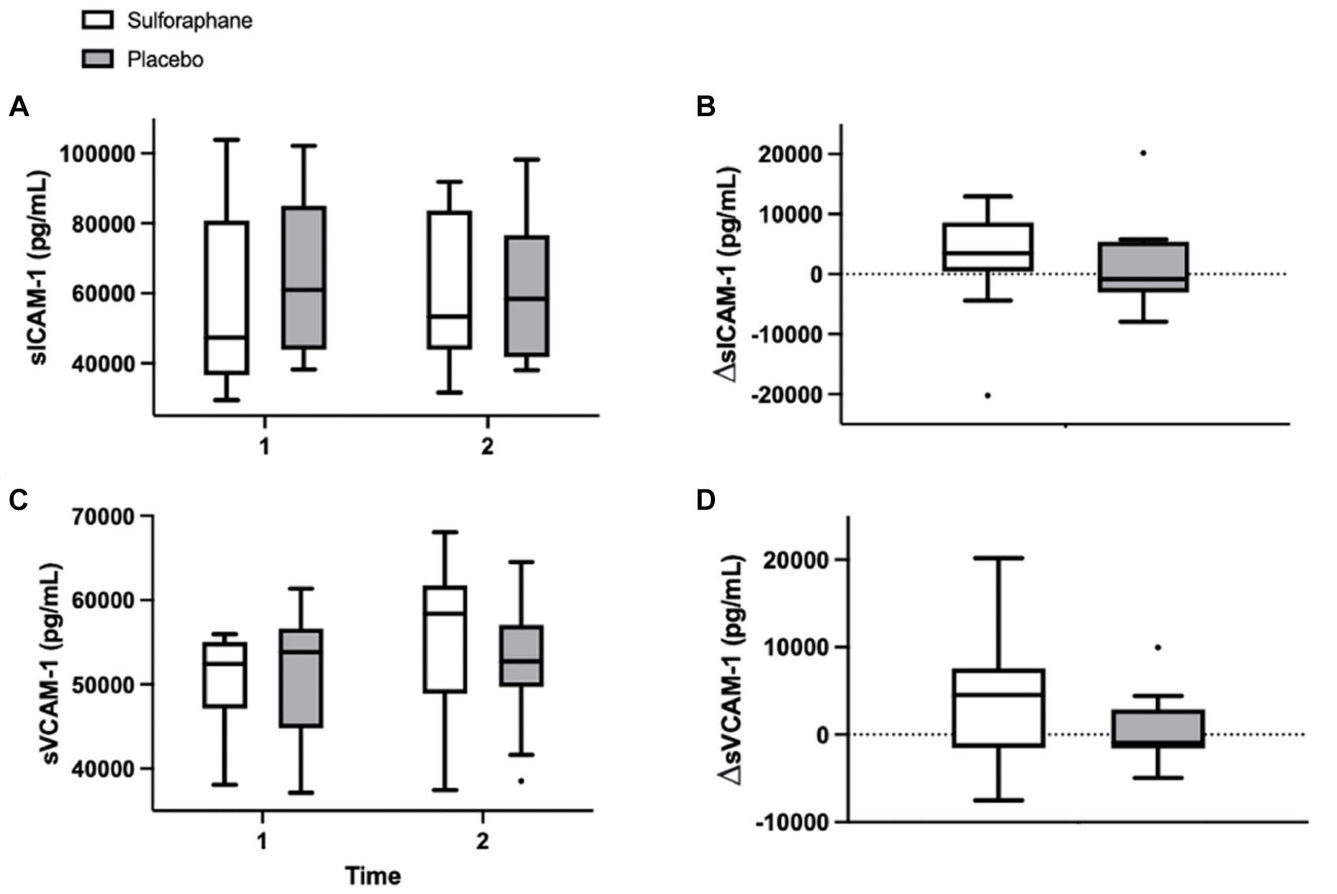
Plasma concentrations of sICAM-1 (pg/mL) and sVCAM-1 (pg/mL) in the sulforaphane and placebo groups before, after, and the changes during the PhenFlex challenge **(A-D)**. Data are presented as boxplots [median, interquartile range, outliers (circles)]. Timepoints: 1—before administration of PhenFlex (90 min); 2—2 h after PhenFlex (210 min). Comparison between timepoints in sulforaphane/placebo.

The single serving of sulforaphane or placebo induced no significant changes in concentrations of sICAM-1 and sVCAM-1 in healthy participants before and 2 h after the PhenFlex challenge. A general trend was seen in the enhancement of endothelial activation in the sulforaphane group, however not significant; sICAM-1 (sulforaphane 1.5 ± 10.1 vs. placebo 3.1 ± 7.8 ng/mL, *p* = 0.696) and sVCAM-1 (sulforaphane 3.1 ± 5.2 vs. placebo 0.9 ± 4.5 ng/mL, *p* = 0.431; Table 2; Figure 3).

**Table 2:** The plasma concentrations of sICAM-1, sVCAM-1, hs-CRP, Adiponectin, CCL-2, and IL-10 before (min 90) and after (min 210) the PhenFlex challenge, Median (IQR).

Inflammatory biomarker		Sulforaphane		Placebo	
sICAM-1^*^ (ng/mL)	Before	56.6 ± 25.6	^#^ *p = 0.428*	63.9 ± 23.3	^#^ *p = 0.513*
	After	59.0 ± 22.3		65.8 ± 20.0	
∆ sICAM-1^*^ (ng/mL)		1.5 ± 10.1		3.1 ± 7.8	^##^*p =* 0.696
sVCAM-1^*^ (ng/mL)	Before	50.5 ± 5.6	^#^ *p = 0.128*	51.5 ± 7.9	^#^ *p = 0.659*
	After	54.5 ± 10.2		52.1 ± 7.9	
∆ sVCAM-1^*^ (ng/mL)		3.1 ± 5.2		0.9 ± 4.5	^##^*p =* 0.431
hs-CRP^*^ (ng/mL)	Before	50.9 ± 9.0	^#^ *p = 0.277*	53.4 ± 10.8	^#^ *p = 0.466*
	After	52.4 ± 8.7		52.7 ± 10.7	
∆ hs-CRP^*^ (ng/mL)		2.2 ± 4.3		−0.5 ± 2.9	^##^*p =* 0.275
Adiponectin^^^ (ng/mL)	Before	50.4 (6.9)	^#^ *p = 0.799*	51.1 (0.8)	^#^ *p = 0.767*
	After	50.9 (3.7)		52.5 (3.8)	
∆ Adiponectin^^^ (ng/mL)		0.5 (4.1)		0.5 (3.7)	*^##^p =* 0.779
CCL-2 (pg/mL)	Before	16.9 (25.6)	^#^ *p = 0.314*	18.2 (24.2)	^#^ *p = 0.374*
	After	19.1 (24.1)		18.3 (23.5)	
∆ CCL-2 (pg/mL)		1.9 (3.3)		0.0 (4.8)	^##^ *p = 0.017*
IL-10^^^ (pg/mL)	Before	55.7 (213.4)	^#^ *p = 0.893*	51.4 (178.8)	^#^ *p = 0.225*
	After	51.2 (231.9)		56.1 (184.1)	
∆ IL-10^^^ (pg/mL)		−0.6 (26.3)		4.4 (5.1)	^##^*p =* 0.715

Significance for ^#^within and ^##^between group comparison; ^^^Anti-inflammatory biomarker; ^*^Normally distributed (mean ± SD).

### 3.3 The effect of sulforaphane on inflammatory biomarkers

**Figure 4:**
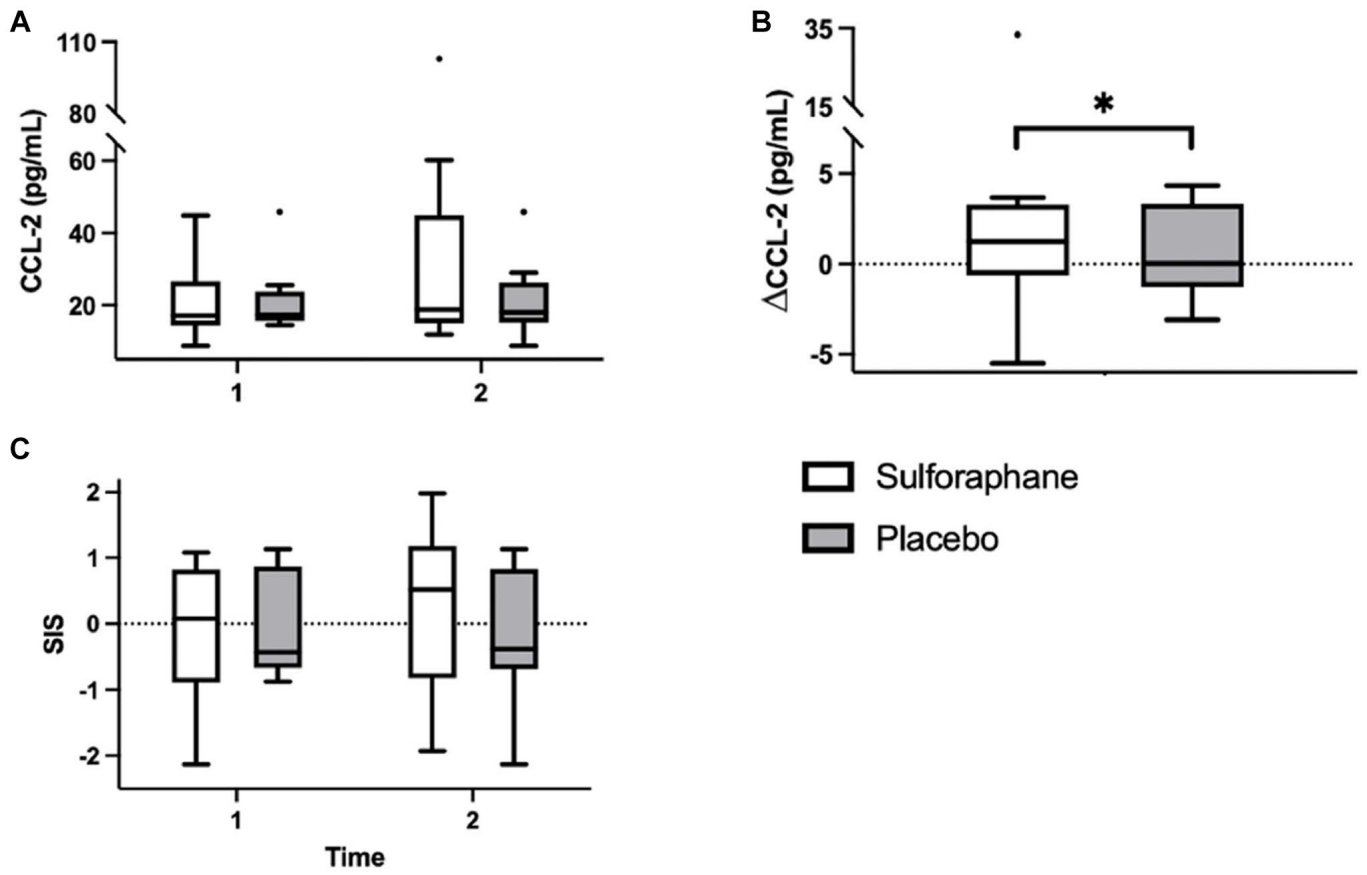
Plasma concentrations of CCL-2 (pg/mL) in the sulforaphane and placebo groups before, after, and the changes during the PhenFlex challenge **(A,B)**. The systemic low-grade inflammation score (SIS) in the sulforaphane and placebo before and after the PhenFlex challenge **(C)**. Data are presented as boxplots [median, interquartile range, outliers (circles)]. Timepoints: 1—before administration of PhenFlex (90 min); 2—2 h after PhenFlex (210 min). Comparison between timepoints in sulforaphane/placebo, *p < 0.05.

Eleven inflammatory biomarkers, sICAM-1, sVCAM-1, IL-6, TNF-α, hs-CRP, adiponectin, IL-12 p70, CCL-2, IL-10, IL-1β, and IL-8, were measured in plasma before and 2 h after the PhenFlex challenge. Levels of sICAM-1, sVCAM-1, hs-CRP, adiponectin, CCL-2, and IL-10 and changes are listed in Table 2. Changes in CCL-2, measured as differential concentrations before and 2 h after caloric load, showed a significant change between groups, with sulforaphane causing a significant increase in this biomarker compared to placebo [1.9 (3.3) vs. 0.0 (4.8) pg./mL, *p* = 0.017; Figure 4]. Changes in sICAM-1, sVCAM-1, hs-CRP (sulforaphane 2.2 ± 4.3 vs. placebo −0.5 ± 2.9 pg./mL, *p* = 0.275), and IL-10 [sulforaphane −0.6 (26.3) vs. placebo 4.4 (5.1) pg./mL, *p* = 0.715] revealed an overall slight and statistically non-significant pro-inflammatory effect of sulforaphane (Table 2). No detectable levels of IL-6, TNF-α, IL-12, IL-1β, and IL-8 were quantified.

### 3.4 The effect of sulforaphane on the systemic low-grade inflammation score

Aside from CCL-2, a more robust change was observed with the integration of the individual biomarkers in the composite systemic low-grade inflammation score (Table 3; Figure 4). In the sulforaphane group the composite score revealed a pro-inflammatory trend after caloric challenge [−0.092 (1.06) before vs. after 0.018 (1.06), *p* = 0.087] which was less prominent in the placebo group [−0.001 (0.81) before vs. after 0.014 (0.81), *p* = 0.251].

**Table 3:** The systemic low-grade inflammation score (SIS) before (min 90) and after (min 210) the PhenFlex challenge, Mean ± SD.

Systemic low-grade inflammation Score (SIS)		Sulforaphane		Placebo	
SIS	Before	−0.092 ± 1.06	^#^*p =* 0.087	−0.001 ± 0.81	^#^*p =* 0.251
	After	0.018 ± 1.06		0.014 ± 0.81	

^#^Significance for within group comparison.

Before the PhenFlex challenge, no significant correlations between inflammation biomarkers and demographic, anthropometric, and lifestyle data were observed, with the exemption of IL-10 and smoking (*p* = 0.042), and the number of cigarettes per day (p = 0.042). Moreover, moderate strength correlations between the systemic low-grade inflammation score and fat percentage (*r_s_* = −0.551, *p* = 0.079) and the number of cigarettes per day (*r_s_* = 0.557, *p* = 0.075) were observed.

### 3.5 The effect of sulforaphane on glucose and lipid metabolism during caloric overload

The single serving of sulforaphane or placebo induced no significant changes in concentrations of glucose and lipoprotein A in healthy participants before and 2 h after the PhenFlex challenge (Table 4). Before the PhenFlex challenge, fasting glucose levels correlated negatively with age (*p* = 0.003), BMI (*p* = 0.046) and fat percentage (*p* = 0.034), and positively with adiponectin concentrations (*p* = 0.015). Fasting lipoprotein A concentrations correlated positively with CCL-2 (*p* = 0.049) and IL-10 levels (*p* = 0.005; Table 5). Moreover, administration of sulforaphane caused changes in glucose levels in response to the caloric load which correlated positively with changes in sICAM-1 (*p* = 0.006), adiponectin (*p* = 0.048) and lipoprotein A (p = 0.005). Changes in lipoprotein A concentrations in response to the challenge after sulforaphane administration correlated positively with changes in sICAM-1 levels (*p* = 0.004). In the placebo group, changes in glucose levels in response to caloric loading only negatively correlated with changes in IL-10 (*p* < 0.001; Table 6).

**Table 4:** The plasma concentrations of glucose and lipoprotein A before (min 90) and after (min 210) the PhenFlex challenge, Mean ± SD.

Parameter		Sulforaphane		Placebo	
Glucose^*^ (mg/dL)	Before	73.8 ± 7.8	*^#^p = 0.363*	76.8 ± 5.2	^#^ *p = 0.133*
	After	66.8 ± 11.5		67.0 ± 16.7	
∆ Glucose^*^ (mg/dL)		−6.9 ± 17.8		−9.8 ± 18.5	^##^*p =* 0.589
Lipoprotein A^*^ (μg/mL)	Before	166 ± 128	^#^ *p = 0.214*	189 ± 144	^#^ *p = 0.269*
	After	172 ± 132		199 ± 130	
∆ Lipoprotein A^*^ (μg/mL)		3.8 ± 14.6		10.7 ± 26.3	^##^*p =* 0.580

Significance for ^#^within and ^##^between group comparison; ^*^Normally distributed.

**Table 5:** Univariate correlates of demographic parameters with fasting metabolic and inflammatory parameters.

Parameter		Demographic					Inflammatory		
		Age	BMI	FP	Smo	Cig	Adi	CCL-2	IL-10
Metabolic	Glucose	−0.81	−0.61	−0.64	-	-	0.71	-	-
	Lp(a)	-	-	-	-	-	-	0.61	0.94
Inflammatory	IL-10	-	-	-	−0.83	−0.83			

Variables were correlated using Pearson (normally distributed parameters) or Spearman’s rank (non-normally distributed) correlation. Only significant correlations are shown. FP, Fat percentage; Smo, Smoking; Cig, Cigarettes per day; Lp(a), Lipoprotein A; Adi, Adiponectin; IL-10, Interleukin-10; CCL-2, Chemokine ligand 2; SIS, Systemic low-grade inflammation score.

**Table 6:** Univariate correlates of changes in metabolic parameters (glucose and lipoprotein A) with inflammatory parameters during the PhenFlex challenge.

	Sulforaphane			Placebo
Parameter	sICAM-1	Adi	Lp(a)	IL-10
Glucose	0.79	0.64	0.81	−1.00
Lipoprotein A	0.82	-	n/a	-

Variables were correlated using Pearson (normally distributed parameters) or Spearman’s rank (non-normally distributed) correlation. Only significant correlations are shown. Lp(a), Lipoprotein A; Adi, Adiponectin.

## 4 Discussion

### 4.1 The PhenFlex challenge did not unbalance endothelial homeostasis in young healthy participants

In the present study, metabolic overload did not significantly affect plasma adhesion marker levels in healthy participants, as measured 2 h after caloric overload. In contrast, previous research has shown that a single administration of the PhenFlex challenge increased the levels of sVCAM-1 and sICAM-1 after 2 h in healthy volunteers (18). Additionally, Derosa et al. demonstrated significant increases in these plasma adhesion markers within 2 h after an oral glucose tolerance test (OGTT) (63). A possible explanation for the lack of effect found on these markers in our study is the fact that the subjects in the current study were given whole food products (sprouts of broccoli or pea) before undergoing the challenge. Both products contain retinol, vitamin E and ascorbic acid, which could have counteracted the expected transient disruption of post-exposure endothelial homeostasis observed in other studies. This hypothesis is supported by findings of Nappo et al., who showed that supplementation with vitamin C and E prevented an increase in sICAM-1 and sVCAM-1 in healthy middle-aged subjects after a high-fat meal (64). Furthermore, Rubin et al. found no changes in plasma adhesion markers in young participants (25 years on average), after a standardized lipid-rich meal which contained retinol (65). Thus, in this study, the effect of sulforaphane on endothelial homeostasis may have been influenced by the other nutrients present in the whole food product. Nonetheless, the associations between metabolic parameters and inflammatory biomarkers during the PhenFlex challenge, particularly between plasma sICAM-1, and glucose and lipoprotein A concentrations in the sulforaphane group, provided relevant information on the modulation of endothelial function and metabolic homeostasis by sulforaphane in response to a high-glucose, high-fat product. Consistent with our findings, Chen et al. demonstrated significant relationships between the plasma glucose and insulin responses to an OGTT and plasma sICAM-1 concentrations in healthy participants (66). The fact that these correlations were not observed in the pea sprouts group (placebo) supports the hypothesis that the other bioactive compounds in the whole food products may have blunted the transient disruption of post-exposure homeostasis expected after caloric load. This is also revealed in part by the only correlation between circulating IL-10 and plasma glucose. The presence of other nutrients in broccoli sprouts may have interfered with the strong effects of sulforaphane, which, however, were still evident as more correlations were shown in this group.

### 4.2 An integrative measure to investigate the pleiotropic effects of phytonutrients is superior to single biomarkers

In this study, sulforaphane facilitated the development of a mild pro-inflammatory state during caloric challenge, as evidenced by a moderate increase in sICAM-1, sVCAM-1, hs-CRP, CCL-2 and decrease in IL-10. The effects of dietary intervention on chronic inflammation in other studies that used the PhenFlex challenge are inconsistent (62, 67). Kim et al. examined the effect of a single-intake microencapsulated garlic powder and/or tomato extract in healthy male smokers during the metabolic challenge. Consumption of tomato extract elicited a differential response, increasing CCL-2 and decreasing sVCAM-1 6 h after PhenFlex compared to placebo. When garlic powder was consumed, IL-13 levels decreased after 2 h and IL-1α increased 6 h after the challenge, indicating a pro-inflammatory effect of the food product. The combination of interventions elicited a mixed response, with IL-10 and CCL-7 being reduced 6 h after the metabolic challenge (67). Hoevenaars et al. investigated the effects of a 12-week whole grain wheat (WGW) intervention compared to refined wheat (RW) and observed increased CRP, IL-6, IL-8, and decreased IL-1β in RW and decreased CRP, serum amyloid A, IL-8, and IL-10 in WGW, indicating pro- and anti-inflammatory effects in respective groups (62). These inconclusive results highlight the importance of implementing integrative outcome measures to unravel the subtle, pleiotropic effects of phytonutrients. As an illustration, the study of Weseler et al. testing the effects of grape seed extract on multiple biomarkers reflecting vascular health integrated them into a vascular health index, which unveiled an improvement in overall vascular health from flavanols, which was less clear from the analysis of individual outcomes (13, 16). In addition, previous cross-sectional studies evaluated CLGI through an index that pools multiple indicators to provide a better overall picture of the synergistic changes of inflammatory biomarkers (51, 68–72).

To the best of our knowledge, this experimental study is the first to examine the effects of phytonutrients on calorie-induced inflammation as measured by a composite scoring system. Intriguingly, the score more accurately reflected the pro-inflammatory effect of broccoli sprouts than single biomarkers during phasic response. In addition, the relationships between risk factors for the development of NCDs such as high visceral fat and smoking, and inflammation became more apparent through the use of the score. Specifically, fat percentage and smoking showed a moderate inverse relationship with the score. Using the systemic low-grade inflammation score also led to another finding; five of the 11 inflammatory biomarkers were undetectable in the blood of our young and healthy population. These findings suggest that even a well-characterized scoring system may show limitations. For future research, assessing the health status or risk profile of the test population and adjusting the scoring system could be beneficial. The six biomarkers detectable under basal conditions in our study may be more suitable for challenge testing in young healthy subjects (19, 36, 73–76).

### 4.3 Metabolic challenge studies may reveal the beneficial effects of phytonutrients in multiple ways depending on mechanism of action in the body

**Figure 5:**
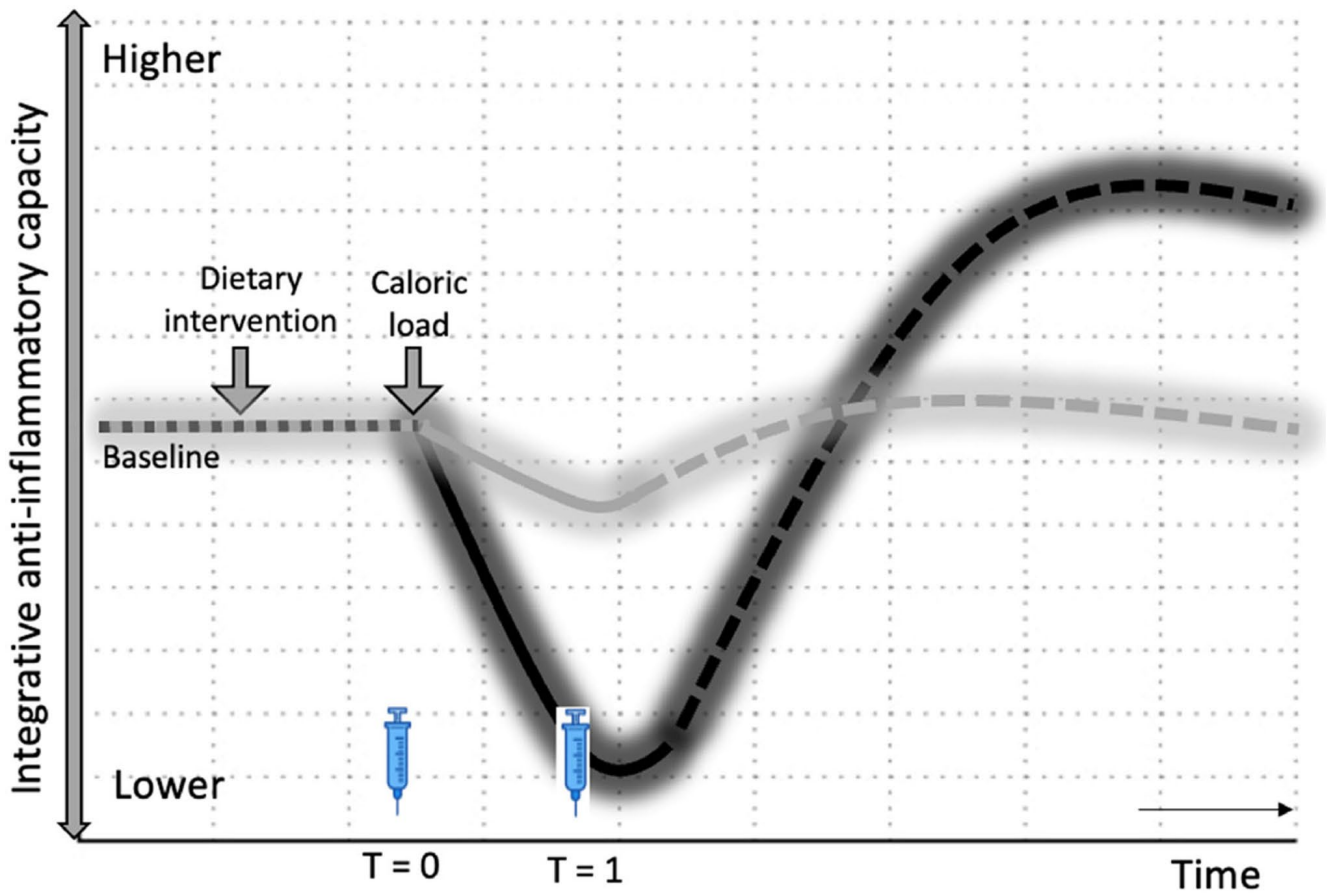
Hormesis hypothesis on health effects of fruits and vegetables. Changes in integrative anti-inflammatory capacity in response to intervention, followed by caloric load. The dotted lines represent the expected sustained effects on integrative anti-inflammatory potential through an increase in endogenous antioxidants via Nrf2 activation by hormetins. Black: Broccoli sprouts (with sulforaphane); Gray: Pea sprouts (without sulforaphane); T0 and T1—time points of blood sampling.

Over the past few decades, research has increasingly focused on antioxidants as the main health-promoting compounds in fruits and vegetables, leading to a gigantic array of antioxidant supplements on the market today (5–7, 12, 77–79). However, initial excitement regarding the potential health benefits of antioxidants failed to be confirmed by clinical evidence (12). There is quite a bit of debate about whether supplementing with antioxidants is healthy, ineffective, or even harmful (5, 11, 12, 22, 28, 36, 77, 78, 80–86). So far, whole foods and fresh produce have not shown a clear protective effect against the PhenFlex challenge (62, 67). In fact, dietary interventions high in hormetins facilitated the development of a mild pro-inflammatory state during caloric overload, e.g., broccoli sprouts and garlic extract (67). We hypothesize that this moderate pro-inflammatory state in the sprouts containing sulforaphane may be due to the initial pro-oxidative action (to activate Nrf2) of hormetins present in fresh produce. As a result, the exogenous antioxidant capacity of the direct antioxidants present in both sprouts is blunted in the broccoli sprouts compared to the pea sprouts. This, in turn, led to a reduced initial integrative anti-inflammatory capacity against the caloric overload demonstrated by the broccoli sprouts compared to the placebo. However, we speculate that because sulforaphane also enhances endogenous antioxidant defenses via Nrf2 activation at a later stage, whole foods that increase both exogenous and endogenous antioxidants may have more significant effects on phenotypic flexibility (Figure 5). This may explain why supplementation of direct antioxidants such as ascorbic acid and vitamin E attenuates the metabolic stress of caloric loads (64, 65), while hormetins in fresh produce, e.g., sulforaphane, diallyl sulfide, withaferin A and rutin, induce a mild pro-inflammatory effect via activation of the Nrf2-pathway (38, 49, 50, 67, 87). We hypothesize that the health effects of fruit and vegetable consumption are due to the wide variety of bioactive compounds in the food matrices and the synergy between the different mechanisms of action of these phytonutrients in the body, rather than just antioxidants (36). One aspect of synergy may be a buffering effect (88, 89). The effect of a large intake of a given nutrient may vary depending on whether it is taken in concentrated form or as part of a food matrix, e.g., the matrix may slow down the absorption of the nutrient, which lowers the likelihood of a bolus effect (89).

The limitations of our study include a small sample size and a short observation period. A longer time of observation (6, 8, 12, or even 24 h) could have helped to demonstrate that the increase in inflammatory activity caused by sulforaphane represents the initial part of the hormetic response. In fact, previous research, conducted with larger sample sizes, has demonstrated the sustained anti-inflammatory effects of sulforaphane (56, 90). At the same time, biotechnological advancements that allow continuous monitoring of certain functions (e.g., glucose) and innovative designs (e.g., n-of-1 trials) may enable more accurate research into personalized nutrition strategies in the future (91–94). Learning more about the interplay between phytonutrients may eventually reveal whether “an apple a day can keep the doctor away”—at least for a while.

## 5 Conclusion

This study has shown that the subtle and pleiotropic effects of phytonutrients can be studied in a short time by challenging the resilience and efficacy of adaptive mechanisms of healthy participants. Broccoli sprouts containing sulforaphane facilitated the development of a mild pro-inflammatory state during the caloric challenge, which suggests the onset of a hormetic response and became more evident when applying integrative outcome measures. The multifaceted approach allowed for more accurate quantification of the effects of phytonutrients in relation to inflammation and metabolic processes. Considering innovative integrative research approaches (e.g., composite scores, wearables, n-of-1 designs) would enhance our understanding of the hormetic principles of phytonutrients and stimulate research into the health effects of food.

## Data availability statement

The raw data supporting the conclusions of this article will be made available by the authors, without undue reservation.

## Ethics statement

The studies involving humans were approved by Medical Ethics Review Committee of Maastricht University Medical Centre+ (MUMC+) and Maastricht University, Maastricht, the Netherlands. The studies were conducted in accordance with the local legislation and institutional requirements. The participants provided their written informed consent to participate in this study.

## Author contributions

HS: conceptualization, investigation, formal analysis, visualization, and writing—original draft. AV: investigation and formal analysis. FO: methodology and writing—review and editing. HP and FT: writing—review and editing. ABa: conceptualization, writing—review and editing, and supervision. KS: conceptualization, data curation, methodology, visualization, writing—review and editing, and supervision. ABo: conceptualization, writing—review and editing, funding acquisition, and supervision. All authors contributed to the article and approved the submitted version.
