# Supplementary material for: Sulforaphane as a potential modifier of calorie-induced inflammation: a double-blind, placebo-controlled, crossover trial
Source: Front Nutr. 2023 Nov 28;10:1245355. doi: 10.3389/fnut.2023.1245355 (PMC10713815; doi:10.3389/fnut.2023.1245355)
Supplement: Supplementary file 1 [file Table_1.docx]

**Supplementary Table 1: DuoSet ELISA kits from R&D systems used for detection of biomarkers in the plasma samples**

| sVCAM-1 | Human VCAM-1/CD106 DuoSet ELISA (R&D systems, #DY809-05) |
| --- | --- |
| sICAM-1 | Human ICAM-1/CD54 DuoSet ELISA (R&D systems, #DY720-05) |
| IL-1β | Human IL-1 beta/IL-1F2 DuoSet ELISA (R&D systems, #DY201-05) |
| IL-6 | Human IL-6 DuoSet ELISA (R&D systems, #DY206-05) |
| TNF-α | Human TNF-alpha DuoSet ELISA (R&D systems, #DY210-05) |
| CCL-2 | Human CCL2/MCP-1 DuoSet ELISA (R&D systems, #DY279-05) |
| IL-8 | Human IL-8/CXCL8 DuoSet ELISA (R&D systems, #DY208-05) |
| IL-10 | Human IL-10 DuoSet ELISA (R&D systems, #DY217B-05) |
| Adiponectin | Human Adiponectin/Acrp30 DuoSet ELISA (R&D systems, #DY1065-05) |
| Hs-CRP | Human C-Reactive Protein/CRP DuoSet ELISA (R&D systems, #DY1707) |
| IL-12 p70 | Human IL-12 p70 DuoSet ELISA (R&D systems, #DY1270-05) |
